# Liquid biopsy for the assessment of adrenal cancer heterogeneity: where do we stand?

**DOI:** 10.1007/s12020-022-03066-z

**Published:** 2022-05-13

**Authors:** Pál Perge, Gábor Nyirő, Bálint Vékony, Peter Igaz

**Affiliations:** 1 Department of Internal Medicine and Oncology, Faculty of Medicine, Semmelweis University, H-1083 Budapest, Hungary; 2 Department of Endocrinology, ENS@T Research Center of Excellence, Faculty of Medicine, Semmelweis University, H-1083 Budapest, Hungary; 3 MTA-SE Molecular Medicine Research Group, Eötvös Loránd Research Network, H-1083 Budapest, Hungary; 4 Institute of Laboratory Medicine, Faculty of Medicine, Semmelweis University, H-1089 Budapest, Hungary

**Keywords:** Adrenocortical cancer, liquid biopsy, circulating tumor cell, circulating cell-free DNA, microRNA, temporal heterogeneity

## Abstract

Almost 10 years have passed since the first attempts of liquid biopsy aimed at the characterisation of tumor cells present in the bloodstream from a regular sample of peripheral blood were performed. Liquid biopsy has been used to characterise tumor heterogeneity in various types of solid tumors including adrenocortical carcinoma. The development of molecular biology, genetics, and methodological advances such as digital PCR and next-generation sequencing allowed us to use besides circulating tumor cells a variety of circulating cell-free nucleic acids, DNAs, RNAs and microRNAs secreted by tumors into blood and other body fluids as specific molecular markers. These markers are used for diagnosis, to check tumor development, selecting efficient therapies, therapy monitoring and even possess prognostic power. In adrenocortical carcinoma, there are some studies reporting analysis of circulating tumor cells, circulating cell free DNA and microRNAs for assessing tumor heterogeneity. Among microRNAs, *hsa-miR-483-5p* seems to be the most important player. Combined with other microRNAs like *hsa-miR-195*, their expression correlates with recurrence-free survival. Most studies support the applicability of liquid biopsy for assessing temporal tumor heterogeneity (i.e. tumor progression) in adrenocortical cancer. In this mini-review, the available findings of liquid biopsy for assessing tumor heterogeneity in adrenocortical cancer are presented.

## A short overview of tumor heterogeneity

Tumor heterogeneity is the attribute of malignant tumors which describes that different tumor cells can show different genetic or phenotypic properties. This can present for example as differing morphological properties, gene expression profiles, metabolic discrepancies and higher proliferative and metastatic potential [1]. This phenomenon can manifest itself between tumors of a singular origin, in other words, metastases, called inter-tumor heterogeneity, or within a single tumor, called intra-tumor heterogeneity. The exact mechanism of this phenomenon is unknown, though currently, there are two theories as to how it might happen, the cancer stem cell model and the clonal selection model, which are not mutually exclusive, meaning they can both contribute to heterogeneity [2]. Tumor heterogeneity has been observed in a multitude of malignancies, e.g. breast, colon, brain and hematologic neoplasms [3], and also adrenocortical carcinoma [4].

Genomic instability plays a major role in tumor heterogeneity. It ranges in magnitude from single nucleotide variations to whole genome doubling, and can be caused by exposures to DNA altering agents, dysfunction of DNA repair and replication mechanisms [5]. Microsatellite instability is known to be a driving factor in different tumors, e.g. colorectal cancer [6]. Certain medications, e.g. paradoxically chemotherapies used to treat cancers, can increase the genetic instability of tumors, thus contributing to the clonal selection process and disease recurrence [7].

According to the cancer stem cell model, only a small subset of tumor cells is capable of proliferation and the difference in tumor cells is based on which stem cell they originated from. The clonal selection hypothesis [8] states that malignancy begins in a stochastic manner, by changes induced in a normal cell gradually leading to neoplastic proliferation. Subsequent evolutional pressures then diversify the genetic material, leading to different subpopulations all originating from the original tumor. [9].

Tumor microenvironment (TME) can provide an additional source of intra-tumor heterogeneity. TME is a playground where non-cancerous cells can develop interactions with tumor cells, including tumor stroma, proliferating tumor cells, immune cells and blood vessels. These interactions create a complex signaling network influencing the fate of individual cells [10, 11]. The immune system fights tumor development but it is also able to promote tumor growth. Immunoediting adds a new dynamic level to tumor heterogeneity and provides a new set of accessible biomarkers [12, 13].

The spectrum of heterogeneity can be described in two forms, spatial and temporal. Spatial heterogeneity means that different genetic and phenotypic properties are found within or in different metastases originating from the tumor. Temporal heterogeneity is the dynamic variation of the same tumor over time. Both can be observed at the same time, and both contribute to the diminishing effectiveness of treatments and relapses [14].

Understanding tumor heterogeneity is pivotal for future treatments of malignancies, as it is the driving force behind metastasis, relapse, and treatment resistance. Heterogeneity can be measured by molecular biology methods, including genetic sequencing. The invention of high throughput methods made this task significantly easier [15].

To examine the heterogeneity of a metastatic neoplasm, multiple samples should be taken from multiple tumors and for an optimal result at multiple intervals in time. This process would require the patient to undergo multiple biopsies, which is difficult and cumbersome, moreover repeated traditional biopsies might contribute to further metastasizing by physically dispersing tumor cells. A new option is liquid biopsy, which is the process of sampling liquid biological matter with tumor-specific molecular pieces of information.

In this review article, we present data on the potential use of liquid biopsy in adrenocortical cancer for the assessment of tumor heterogeneity. The available few studies target temporal heterogeneity (tumor progression).

## Adrenocortical carcinoma

Adrenocortical tumors (ACT) are common, and their prevalence rises with age [16]. The majority of these neoplasms is represented by benign, hormonally inactive adrenocortical adenomas (ACA). Adrenocortical carcinoma (ACC) is a rare cancer, with an incidence of approximately 0.7–2 annual cases per million population [17]. ACC is an aggressive neoplasm with poor prognosis according to the low 5-year survival rate below 30% in advanced stages [18, 19].

Imaging is pivotal for the follow-up of adrenocortical cancer, but blood-borne tumor markers of progression would be needed, as well.

## Liquid biopsy

Liquid biopsy (LB) at its introduction in 2013, referred to the analysis of circulating tumor cells (CTC) in the blood of cancer patients [20]. Later on, it was extended to include pieces of DNA from tumor cells contained in blood and other body fluids. LB’s great utility was seen as an easy repeatable non-invasive method compared to tissue biopsy used in the diagnosis, prognosis, treatment and follow-up of patients with diverse neoplasms [21]. With the development of methodology (including next-generation sequencing (NGS)), diverse nucleic acids became also available for analysis (cell-free nucleic acids (cfNA)), including circulating cell free DNA (cfDNA) and circulating tumor DNA (ctDNA), coding and non-coding RNAs such as messenger RNA transcripts (cfRNA: cell free RNA, mRNA), and noncoding RNAs (ncRNAs) like rRNAs (ribosomal RNA), tRNAs (transferRNA), long non-coding RNAs (lncRNAs) and microRNAs (miRNA) [22]. These cfNAs can be free circulating, attached to carrier molecules or enveloped in extracellular vesicules (EV) [23] and are released into the circulation or other body fluids by most cancer types at different stages of the disease.

## Potential role of CTC, circulating tumor DNA and microRNAs in the management of ACC

### Circulating tumor cells

LB offers a non-invasive possibility for longitudinal monitoring of genetic and phenotypic changes in heterogeneous cancer [24]. Circulating tumor cells (CTCs) are detectable in peripheral blood samples of patients suffering from different solid tumors [25, 26]. The neoplastic CTCs that are circulating freely in blood are derived either from the primary tumor or from the metastases, thus presenting spatial heterogeneity [27]. CTCs could serve as minimally invasive biomarkers for diagnosis, prognosis, recurrence or to estimate treatment efficacy [27, 28].

CTCs are rather rare: 0.1–10 per mL whole blood. Cell stabilization is important and enrichment of the CTCs is necessary for further applications. CTCs can be characterised based on physical and morphological traits (nuclear irregularity, high nuclear/cytoplasmic ratio) and cell surface markers (epithelial markers: epithelial cell adhesion molecule (EpCAM), cytokeratins) [29]. CTC assays lack 100% sensitivity and specificity in cancer detection since other diseases like inflammatory colon disease can give rise to circulating epithelial cells and a biological shift called epithelial-to-mesenchymal transition (EMT). Epithelial cancers change their expression pattern to mesenchymal in disease progression [30, 31]. Compared to individual CTCs, CTC clusters are more difficult to characterise, although they have higher metastatic potential [32, 33]. Prognostic implication of CTCs is high in breast cancer, colorectal cancer and non-small cell lung cancer. A remarkable advantage of CTCs is that they can be cultured in vitro and expanded to perform functional assays [34, 35].

Regarding ACT and CTCs, only two studies involving small cohorts have been published, to date. In the first study [36], 14 ACC and 10 ACA patients were included. CTCs were detected in all patients affected by ACC, but in none of ACA patients [36]. A significant decrease in the number of CTCs was found after surgery, and there was a correlation between tumor size and CTC concentration. Moreover, a statistically significant difference was detected between metastatic and non-metastatic ACC patients.

In a more recent monocentric study from the same resarch group performed on a small cohort of patients affected with ACC CTCs were raised as potential markers of prognosis and overall survival [37]. 68% of ACC patients were found to harbor CTC in this study, and in some patients tumor recurrence was associated with a rapid increase in CTC number.

### Circulating tumor DNA

The revolutionary discovery of cell free DNAs in circulation and its correlation with different type of cancers was published decades ago [38]. From the biological point of view, the most important proportion of cfDNA is the circulating tumor DNA. The sum of ctDNA in blood predominantly depends on the histological type, the tumor stage and the efficacy of anticancer therapy of the tumor [39, 40]. Similarly to CTCs, ctDNAs are mainly originating from the primary tumor and metastases [41, 42]. The detection of ctDNA in asymptomatic patients showed poor results [43, 44]. The amount of ctDNA can reflect the actual tumor burden [45], since higher yield of ctDNA was detectable in advanced stages of different tumors compared to locoregional malignancy [39]. Furthermore, ctDNA can be applied for evaluating intratumor heterogeneity [46]. In several tumors, e.g. colorectal cancer, there are numerous data on the diagnostic potential of ctDNA [47, 48]. Mutations and methylation patterns of ctDNA can also deliver important pieces of diagnostic information [49, 50]. ctDNAs could be utilized even as prognostic markers, since they can predict response for therapy and risk of metastatic recurrence e.g. in breast and rectal cancer [51, 52].

Two preliminary studies examined the potential biomarker role of ctDNA in ACC, to date. Creemers et al. investigated ctDNAs in ACC patients (*n* = 6) and searched for characteristic tumor specific mutations [53]. Tumor-specific ctDNA mutations were detected in only one of three patients with mutations in the primary ACC.

In Garinet’s study, 11 ACC patients were evaluated [54]. CtDNA mutations proving the tumoral origin of DNA were identified only in a subset of patients (*n* = 2). In the background of this phenomenon, it could be hypothesized that only the most agressive ACCs secreted enough ctDNA that could be detected. A correlation with disease progression was found, thus a potential for assessing ACC temporal heterogeneity could be raised. However, in some patients even with high tumor burden, no ctDNA was detected.

McCabe et al. reported an ACC case where ctDNA analysis was used to monitor tumor recurrence aimed at the detection of a somatic *MSH2* deletion that was identified in the ACC tissue as a pathogenic driver mutation. In this particular case, the somatic *MSH2* deletion could not be found during follow-up, and the patient was considered tumor free [55], thus the utility of this approach could not be validated.

In conlusion, despite some promising results, the application of ctDNA in the clinical setting as a potential biomarker in ACC is not clearly established. It might be useful in a subset of patients, but further studies on larger cohorts are needed to prove its potential utility.

### Extracellular microRNAs in ACC for assessing temporal heterogeneity

MicroRNAs (miRNA, miR) are the endogenous mediators of RNA interference [56]. MicroRNAs are very stable, and their expression can be reliably determined in tissue samples, but also in body fluids [57]. Differentially expressed blood-borne miRNAs were uncovered in patients suffering from different tumors [58, 59]. Extracellular miRNAs are detectable in a wide range of body fluids, including saliva, urine and stool [57, 60]. The biological relevance of circulating miRNA is not clarified [61], but they can serve as markers for diagnosis, and prognosis [59]. Their outstanding stability in body fluids enhances their possible applicability even more [62, 63].

Few circulating blood-borne microRNAs have been found to be differentially expressed between benign and malignant ACT. Circulating *hsa-miR-483-5p* is the most consequently observed overexpressed microRNA in ACC [64, 65].

The diagnostic applicability of circulating miRNAs in different studies is rather variable. Overexpressed *hsa-miR-483-5p* and underexpressed *hsa-miR-195* in ACC should be highlighted [66, 67].

The study by Salvianti et al. using a novel real-time PCR approach aimed at the absolute quantification of circulating *hsa-miR-483* and *hsa-miR-483-5p* in plasma samples of patients with ACT should be highlighted [68]. *Hsa-miR-483-5p* was significantly overexpressed in advanced stages of ACC (stages III-IV) compared to early stages (stages I-II). However, there was no significant association between overall survival and *hsa-miR-483-5p*. Circulating *hsa-miR-483-5p* could differentiate recurring and non-recurring ACC. High circulating *hsa-miR-483-5p* expression was associated with significantly shorter recurrence-free and overall survival [69].

In contrast with ctDNA and CTC, circulating miRNA might be detectable in most ACC blood samples, as exemplified by the absolute quantification of *hsa-miR-483-5p* [68]. However, this does not imply that circulating microRNAs are invariably applicable for ACC diagnosis.

Circulating miRNA might also exploited for the monitoring of of anticancer therapy, but there are only published findings made in animal models available to date [70, 71]. In adrenocortical xenograft mouse models, *hsa-miR-483-5p* [70] and the major hypoxamiR *hsa-miR-210* were affected by anti-cancer treatments [71].

**Fig. 1:**
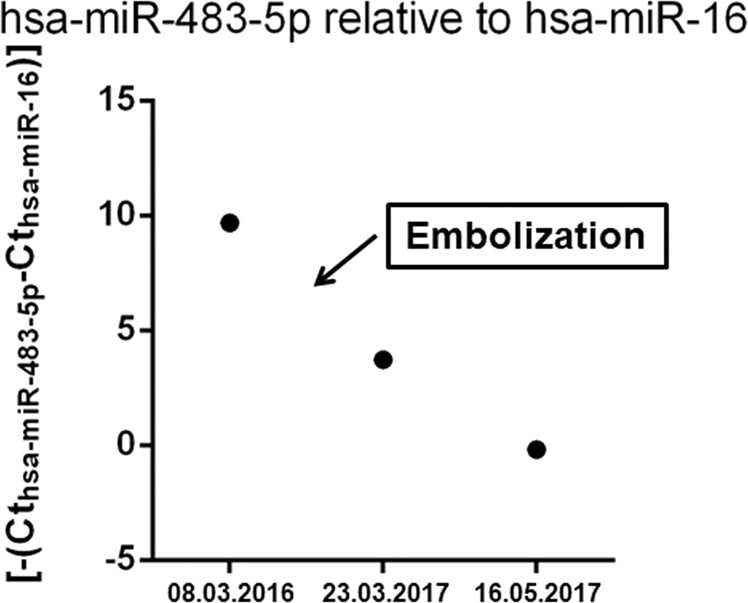
Expression of *hsa-miR-483-5p* measured by RT-qPCR before and after selective arterial embolization treatment of a huge adrenocortical cancer. Normalized to the reference gene *hsa-miR-16*. Results are represented by –dCT (cycle threshold). The clinical features of the case have been presented in [72]

The potential applicability of circulating *hsa-miR-483-5p* as a marker for treatment efficacy monitoring is demonstrated in Fig. 1 [72].

## Conclusions

The investigation of markers of tumor heterogeneity in adrenocortical cancer are in the preliminary phase. Few studies are available including some promising approaches. Circulating tumor cells and some circulating microRNAs appear to be the most promising modalities, but further studies on larger cohorts with uniform methodologies will be needed to assess the applicability of these techniques in the clinical setting.
